# Supplementary material for: From sequence to enzyme mechanism using multi-label machine learning
Source: BMC Bioinformatics. 2014 May 19;15:150. doi: 10.1186/1471-2105-15-150 (PMC4229970; doi:10.1186/1471-2105-15-150)
Supplement: Additional file 2 — Java code of ml2db. Additional file ml2db_code.tar.gz contains the Java source code to run the multi-label machine learning experiments and save the results to database. The code’s Javadoc is included. [file 1471-2105-15-150-S2.zip › additional file 2/ml2db/ml2db/doc/test/ml2db/mulan/predict/PredictionsSaverTest.html]

PredictionsSaverTest


JavaScript is disabled on your browser.


- Overview
- Package
- Class
- Use
- Tree
- Deprecated
- Index
- Help

- Prev Class
- Next Class

- Frames
- No Frames

- All Classes

- Summary:
- Nested |
- Field |
- Constr |
- Method

- Detail:
- Field |
- Constr |
- Method


test.ml2db.mulan.predict

## Class PredictionsSaverTest

- java.lang.Object
- - junit.framework.Assert
  - - junit.framework.TestCase
    - - test.ml2db.mulan.predict.PredictionsSaverTest

- All Implemented Interfaces:
  :   junit.framework.Test

  ---

    

  ```
  public class PredictionsSaverTest
  extends junit.framework.TestCase
  ```

  Before running, make sure the predictions record test is myIsam: ALTER TABLE
  `test`.`prediction\_records` ENGINE = MyISAM ; and instance field is longer:
  ALTER TABLE `test`.`prediction\_records` CHANGE COLUMN `instance` `instance`
  VARCHAR(15) NULL DEFAULT NULL ;
  Train arff: inst1, inst2 etc. att 1, 3, 4, 5 class 1.1.1.1, 2.2.2.2
  Test arff: instA, instB, instC etc. att 2, 3 class 2.2.2.2, 3.3.3.3
  Test data filtered by train: cannot contain att 2, must contain att1, 3, 4, 5
  Types comment: change at eclipse > java > code style > code template > Types

- - ### Constructor Summary

    Constructors

    Constructor and Description

    `PredictionsSaverTest()`
  - ### Method Summary

    Methods

    | Modifier and Type | Method and Description |
    | --- | --- |
    | `static PredictionsSaver` | `getSaver(java.lang.String trainDatasetName, java.lang.String trainProps, java.lang.String testDatasetName, java.lang.String testProps, MulanLearner learner)` |
    | `static PredictionsSaver` | `predictor1TrainOnTrain()` |
    | `void` | `testPredictions()` |
    | `void` | `testPredictorI1I2_vs_I1I2()` |
    | `void` | `testPredictorI3Empty_vs_I1I2()` |
    | `void` | `testPredictorTrainTest()` |

    - ### Methods inherited from class junit.framework.TestCase

      `countTestCases, getName, run, run, runBare, setName, toString`
    - ### Methods inherited from class junit.framework.Assert

      `assertEquals, assertEquals, assertEquals, assertEquals, assertEquals, assertEquals, assertEquals, assertEquals, assertEquals, assertEquals, assertEquals, assertEquals, assertEquals, assertEquals, assertEquals, assertEquals, assertEquals, assertEquals, assertEquals, assertEquals, assertFalse, assertFalse, assertNotNull, assertNotNull, assertNotSame, assertNotSame, assertNull, assertNull, assertSame, assertSame, assertTrue, assertTrue, fail, fail, failNotEquals, failNotSame, failSame, format`
    - ### Methods inherited from class java.lang.Object

      `equals, getClass, hashCode, notify, notifyAll, wait, wait, wait`

- - ### Constructor Detail


    - #### PredictionsSaverTest

      ```
      public PredictionsSaverTest()
      ```
  - ### Method Detail


    - #### testPredictions

      ```
      public void testPredictions()
      ```


    - #### testPredictorI1I2\_vs\_I1I2

      ```
      public void testPredictorI1I2_vs_I1I2()
      ```


    - #### testPredictorI3Empty\_vs\_I1I2

      ```
      public void testPredictorI3Empty_vs_I1I2()
      ```


    - #### testPredictorTrainTest

      ```
      public void testPredictorTrainTest()
      ```


    - #### getSaver

      ```
      public static PredictionsSaver getSaver(java.lang.String trainDatasetName,
                              java.lang.String trainProps,
                              java.lang.String testDatasetName,
                              java.lang.String testProps,
                              MulanLearner learner)
      ```


    - #### predictor1TrainOnTrain

      ```
      public static PredictionsSaver predictor1TrainOnTrain()
      ```


- Overview
- Package
- Class
- Use
- Tree
- Deprecated
- Index
- Help

- Prev Class
- Next Class

- Frames
- No Frames

- All Classes

- Summary:
- Nested |
- Field |
- Constr |
- Method

- Detail:
- Field |
- Constr |
- Method
